# Integrated lipase production and *in situ* biodiesel synthesis in a recombinant *Pichia pastoris* yeast: an efficient dual biocatalytic system composed of cell free enzymes and whole cell catalysts

**DOI:** 10.1186/1754-6834-7-55

**Published:** 2014-04-08

**Authors:** Jinyong Yan, Xianliang Zheng, Lei Du, Shengying Li

**Affiliations:** 1 Key Laboratory of Biofuels, Shandong Provincial Key Laboratory of Energy Genetics, Qingdao Institute of Bioenergy and Bioprocess Technology, Chinese Academy of Sciences, No. 189 Songling Road, Qingdao, Shandong 266101, China

**Keywords:** Integrated biodiesel production, Lipase, *Pichia pastoris*, *Thermomyces lanuginosus*, Waste cooking oils

## Abstract

**Background:**

Lipase-catalyzed biotransformation of acylglycerides or fatty acids into biodiesel via immobilized enzymes or whole cell catalysts has been considered as one of the most promising methods to produce renewable and environmentally friendly alternative liquid fuels, thus being extensively studied so far. In all previously pursued approaches, however, lipase enzymes are prepared in an independent process separated from enzymatic biodiesel production, which would unavoidably increase the cost and energy consumption during industrial manufacture of this cost-sensitive energy product. Therefore, there is an urgent need to develop novel cost-effective biocatalysts and biocatalytic processes with genuine industrial feasibility.

**Result:**

Inspired by the consolidated bioprocessing of lignocellulose to generate bioethanol, an integrated process with coupled lipase production and *in situ* biodiesel synthesis in a recombinant *P. pastoris* yeast was developed in this study. The novel and efficient dual biocatalytic system based on *Thermomyces lanuginosus* lipase took advantage of both cell free enzymes and whole cell catalysts. The extracellular and intracellular lipases of growing yeast cells were simultaneously utilized to produce biodiesel from waste cooking oils *in situ* and in one pot. This integrated system effectively achieved 58% and 72% biodiesel yield via concurrent esterified-transesterified methanolysis and stepwise hydrolysis-esterification at 3:1 molar ratio between methanol and waste cooking oils, respectively. Further increasing the molar ratio of methanol to waste cooking oils to 6:1 led to an 87% biodiesel yield using the stepwise strategy. Both water tolerance and methanol tolerance of this novel system were found to be significantly improved compared to previous non-integrated biodiesel production processes using separately prepared immobilized enzymes or whole cell catalysts.

**Conclusion:**

We have proposed a new concept of integrated biodiesel production. This integrated system couples lipase production to lipase-catalyzed biodiesel synthesis in one pot. The proof-of-concept was established through construction of a recombinant *P. pastoris* yeast strain that was able to grow, overexpress *T. lanuginosus* lipase, and efficiently catalyze biodiesel production from fed waste cooking oils and methanol simultaneously. This simplified single-step process represents a significant advance toward achieving economical production of biodiesel at industrial scale via a ‘green’ biocatalytic route.

## Introduction

Biodiesel comprising long-chain fatty acid methyl esters (FAMEs) prepared from transesterification between acylglycerides and methanol is one of the most promising biofuel alternatives to traditional fossil fuels. Lipase-based enzymatic routes toward biodiesel production hold numerous advantages over chemical methods using alkaline and/or acid catalysts, including environmentally friendly conditions, easy separation of byproduct glycerol, and simultaneous esterification of free fatty acids (FFAs) and transesterification of glycerides, an essential characteristic for biodiesel production from high FFA content waste cooking oils (WCOs) [1].

However, biodiesel derived from chemical transformations still dominates the current global market, mainly due to its lower cost than the equivalent prepared by biocatalytic processes [2]. Because both transformations use a common feedstock, the spending on catalysts becomes the key factor for overall cost. The cost of lipases is determined by the catalytic activity, expression level, and preparation method. Thus, great efforts have been made to obtain a catalytically efficient and cost-effective lipase to compete with the current chemical catalysts used for biodiesel production, especially at large scale.

To date, immobilized lipases and whole cell lipase catalysts have been the most studied biocatalysts for production of biodiesel. For instance, a number of commercial immobilized lipase preparations such as Novozyme 435 and Lipozyme TLIM have been evaluated for this process [3]. Immobilized enzymes have flexible performance on activity and stability, but suffer high preparation cost. Wild-type filamentous fungus *Rhizopus oryzae* containing homogenous cell-bound lipases [4] and recombinant *Aspergillus oryzae* expressing heterologous lipases have been used as whole cell catalysts for biodiesel production [5]. Recombinant *Escherichia coli* cells producing intracellular lipase were employed to catalyze biodiesel formation [6]. *Saccharomyces cerevisiae* and *Pichia pastoris* yeast cells with surface displayed lipases were also tested for the same purpose [7,8]. As recombinant yeast whole cell catalysts in the form of intracellular enzymes, *S. cerevisiae* yeast cells with intracellular expression of *R. oryzae* lipase were engineered to generate biodiesel [9]. Recently, we also constructed *P. pastoris* yeast whole cell catalysts for biodiesel synthesis with intracellular expression of *Thermomyces lanuginosus* lipase (Tll) [10]*.*

**Figure 1:**
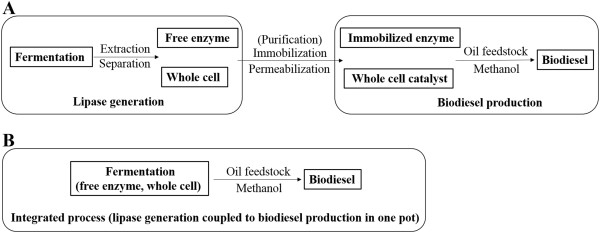
**Two distinct strategies for biodiesel production. (A)** Conventional enzymatic biodiesel production route separating lipase generation and biodiesel production processes. **(B)** Integrated route coupling lipase generation to biodiesel production in one pot as proposed in the present study.

However, in all these practices, immobilized enzymes and whole cell catalysts were prepared in an independent process separated from enzymatic biodiesel production (Figure 1A), which would unavoidably increase the cost and energy consumption during industrial manufacture of this cost-sensitive energy product. Therefore, a simplified integrated bioprocess that combines lipase production and enzymatic biodiesel production into a single stage in one pot is preferred (Figure 1B). Inspired by consolidated bioprocessing of lignocellulose to bioethanol [11], in which cellulase production, cellulose hydrolysis and ethanol fermentation are integrated into a single step for lowering cost and simplifying process, we used *P. pastoris* yeast with expression of Tll as a model system to investigate the feasibility of integrated biodiesel production.

Tll was selected because it has been widely used for biodiesel production, modification of oils, chiral resolution of racemic compounds and other purposes [12]. *P. pastoris* yeast has been proven as an excellent system for expression of foreign proteins. Compared with *S. cerevisiae* yeast, *P. pastoris* can achieve higher cell density, and greater expression level of functional heterologous enzymes due to less hyper-glycosylation [13]. There have been extensive genetic engineering efforts aimed at over-production of diverse lipases in *P. pastoris* yeast [13,14].

In the integrated biodiesel production, the normally separated biocatalyst preparation and enzyme-mediated biotransformation were integrated into a single step, thus bypassing the costly enzyme extraction and preparation. More importantly, this novel process did not compromise the efficiency of biodiesel synthesis, and demonstrated better water and methanol tolerance than the conventional routes. Therefore, it holds great potential to be developed into an industrial biocatalytic system for large-scale biodiesel production.

## Results and discussion

### Expression of lipase in recombinant *P. pastoris* yeast

*P. pastoris* yeast has been shown to be a highly efficient system for secreted expression of various heterologous lipases [13]. In these expression systems, extracellular lipases have been intensively investigated. However, little attention has been paid to the portion of lipases remaining inside yeast cells, leaving an important question to be answered: how much catalytic capacity is discarded during typical preparation of lipase products only using extracellular enzymes?

**Figure 2:**
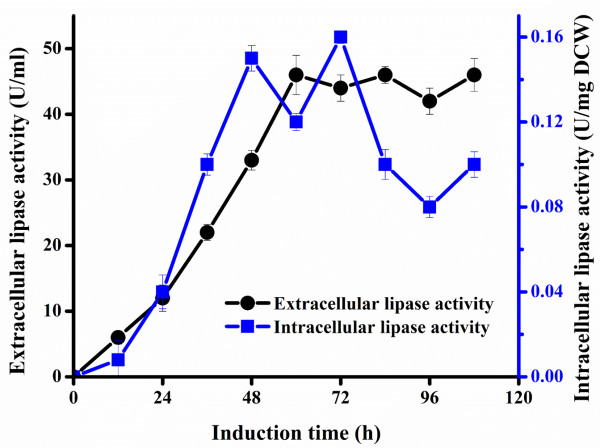
**Time course of the olive oil hydrolyzing activity of cell free *T. lanuginosus *lipase (extracellular) and whole cell catalyst (intracellular) produced by the recombinant *P. pastoris *yeast.** DCW, dry cell weight.

To answer this question, we determined the olive oil hydrolyzing activity of extracellular and intracellular lipases. As expected, the majority of functional Tll enzymes were detected in the supernatant of fermentation culture, with the highest extracellular lipase production reaching 46 U/ml at 60 h (Figure 2). Interestingly, intracellular lipases in the form of whole cell catalysts displayed activity ranging from 0.08 to 0.16 U/mg dry cell weight (DCW) between 36 and 108 h. This result clearly indicated that not all lipases were secreted under the guidance of *α*-factor signal peptide, probably due to the limited capacity of the secretory machinery of *P. pastoris* relative to the high amount of overexpressed Tll. Therefore, the cost for biodiesel production using lipases could be further lowered by taking advantage of the otherwise abandoned intracellular lipase activity.

### Determination of the activity of extracellular and intracellular *T. lanuginosus* lipase for biodiesel formation

**Figure 3:**
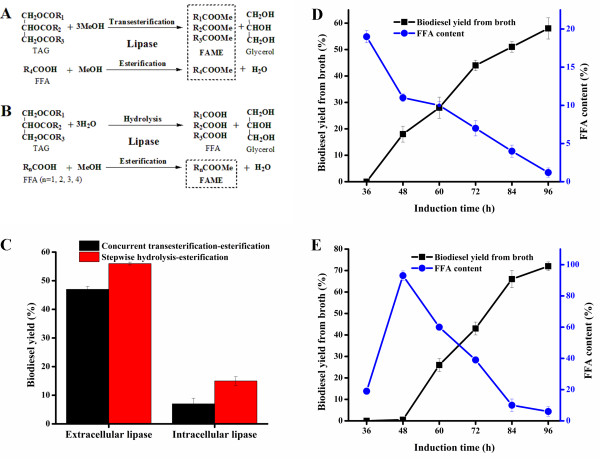
**Biodiesel production systems investigated in this study.** Schemes of biodiesel production by **(A)** concurrent transesterification-esterification process and **(B)** stepwise hydrolysis-esterification process. **(C)** Biodiesel production by separated extracellular and intracellular lipase using different processes. Integrated biodiesel production via **(D)** concurrent esterification-transesterification and **(E)** stepwise hydrolysis-esterification.

Concurrent transesterification-esterification methanolysis and stepwise hydrolysis-esterification are the two major processes for lipase-mediated biodiesel production (Figure 3A, B) [15-20]. Lipase-catalyzed esterification of FFAs is likely to be more efficient than direct transesterification of triglycerides because three acyl groups tethered to the glycerol backbone may cause steric hindrance for enzyme access [21]. Thus, the two-step process consisting of hydrolysis of triglycerides to form FFAs and esterification of the released FFAs (and the pre-containing high content FFAs in WCOs) to generate FAMEs might be more productive than the concurrent esterification and transesterification regarding biodiesel synthesis. To test this hypothesis, we compared these two processes for biodiesel production using separated individual forms (in this section) and in the integrated manner (see the next two sections).

Specifically, the 60 h post-induction supernatant containing 46 U/ml extracellular lipases and corresponding cell pellet with the olive oil hydrolytic activity of 0.12 U/mg DCW (Figure 2) were separated by centrifugation, and used as the liquid cell free lipases and the whole cell lipases for bioconversion of WCOs into biodiesel, respectively. In the concurrent transesterification-esterification methanolysis, the liquid enzymes were able to catalyze biodiesel production with a 47% yield. By contrast, the whole cell catalysts intracellularly maintaining a small portion of lipases afforded a 7% biodiesel yield (Figure 3C). In the stepwise hydrolysis followed by esterification, the liquid cell free lipases and the whole cell lipases gave 56% and 15% biodiesel yields, respectively (Figure 3C). These results not only demonstrate that the stepwise hydrolysis-esterification is more efficient for biodiesel synthesis than the concurrent transesterification-esterification, but also suggest that the intracellular lipases, despite their lower activity, should not be neglected for biodiesel production.

### Integrated biodiesel production via concurrent transesterification and esterification

During routine lipase fermentation, starting from logarithmic phase, 0.5% (v/v, methanol volume relative to culture volume) methanol was added every 12 h as an expression inducer and sole carbon source for induced expression of lipase and growth of *P. pastoris* yeast. In the integrated biodiesel production in this study, Tll-catalyzed *in situ* synthesis of biodiesel was initiated by addition of 10 g WCOs and 2% methanol at 36 h, after which 2% methanol was supplemented an additional three times at 48 h, 60 h, and 72 h, leading to the final 1:3 molar ratio between WCOs and methanol. In addition to being the inducer and carbon source (0.5%, theoretically), extra methanol (1.5%) acted as a reactant for esterification of high content FFAs and transesterification of triglycerides in WCOs. Under this concurrent esterified-transesterified methanolysis, catalyzed *in situ* by both extracellular and intracellular lipases, 58% biodiesel yield was achieved after 60 h reactions (from 36 to 96 h). In the meantime, the FFA content decreased from 19% to 1.2% (Figure 3D). In comparison, separated extracellular and intracellular lipases catalyzed biodiesel formation with yields of 47% and 7% (Figure 3C), respectively. These results clearly indicate that integrated biodiesel production is more efficient than typical biodiesel preparation that uses only secreted lipases. Elimination of the enzyme isolation step also significantly lowers the cost for biodiesel production.

### Integrated biodiesel production via stepwise hydrolysis followed by esterification

In stepwise hydrolysis followed by esterification, 10 g WCOs were added into the induced lipase fermentation culture at 36 h, without supplementation of extra methanol, to initiate only the hydrolysis of triglycerides by Tll. In 12 h hydrolytic reactions, the FFA content went up to 93% from 19%, demonstrating highly effective hydrolysis catalyzed by Tll lipases. It should be noted that phosphate buffer present in the buffered methanol-complex medium (BMMY) was able to balance the decreased pH resulted from released FFAs, thus the pH did not affect the catalytic performance. Subsequently, 2% methanol was added every 12 h from 48 to 84 h to allow esterification of released FFAs. At 96 h, a 72% biodiesel yield was achieved, along with an FFA content decreased to 6% (Figure 3E). Considering that 93% FFAs decreased to 6% but the biodiesel yield was only 72%, the residual 15% FFAs could have been reassembled to glycerol backbone.

Because the total amount of methanol and the reaction time were kept the same in the concurrent transesterification-esterification and the stepwise hydrolysis followed by esterification, we reason that the varied biodiesel yield can be attributed to different reaction processes resulting from the distinct time points for the addition of the reactant methanol. The latter strategy was more effective for *in situ* integrated synthesis of biodiesel than the former (72% versus 58%). In addition to different reaction types (transesterification-esterification versus hydrolysis-esterification), the cell permeability of substrates (acylglycerides versus FFAs) might be another important factor leading to the varied biodiesel yield. Given that FFAs with smaller molecular size could more easily penetrate the cell membranes than larger glycerides, stepwise hydrolysis-esterification would allow more substrates to contact intracellular lipases, consequently giving a higher biodiesel yield than concurrent transesterification and esterification (Additional file 1).

### Effect of water content on integrated biodiesel production via stepwise hydrolysis followed by esterification

**Figure 4:**
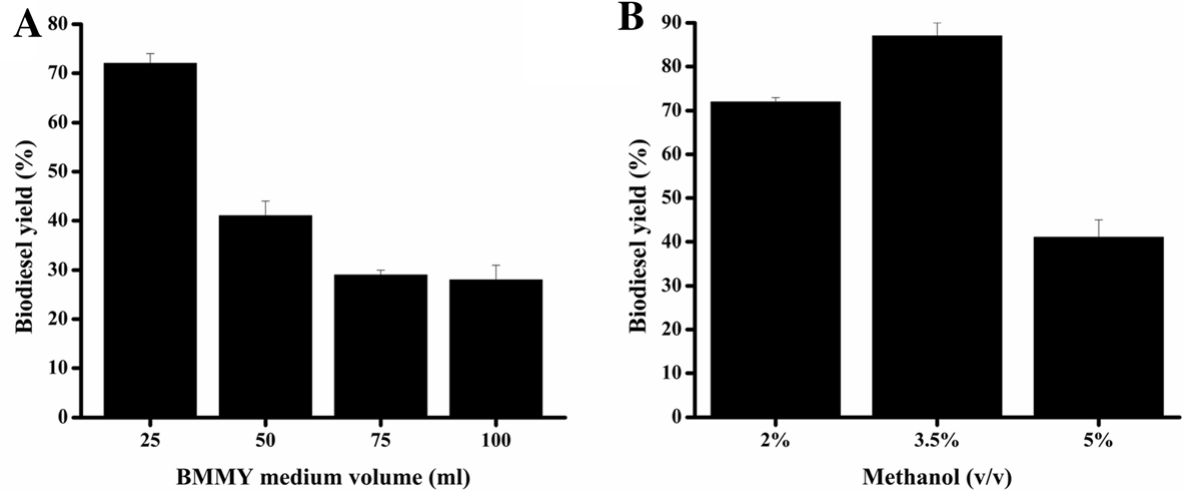
Effect of (A) water content and (B) methanol concentration on integrated biodiesel production via stepwise hydrolysis-esterification.

For recombinant protein production by the methanol utilization plus *P. pastoris* strain, 25 ml buffered complex glycerol medium (BMGY) followed by 100 to 200 ml BMMY medium was generally used in shaking flask fermentation [14]. For the *P. pastoris* integrated biodiesel production system under the stepwise hydrolysis-esterification strategy, the water present in this system is used as a reactant for hydrolysis of triglycerides. However, too much water drives the equilibrium of the reaction towards hydrolysis, not esterification of FFAs [22]. To determine a favorable water content for biodiesel production, the water ratio was controlled by adjusting the BMMY medium volume (25, 50, 75, and 100 ml) to balance the above two aspects. As a result, the highest 72% biodiesel yield was achieved in the lowest volume (25 ml) reaction system (Figure 4A). This suggests that the water content in the integrated system should be limited at a low level provided that the cell growth and hydrolysis of triglycerides is not negatively affected. It should be noted that 25 ml was almost the lowest volume limit of BMMY medium; a lower volume was not viable for cell growth, enzyme expression, and triglyceride hydrolysis because no free water remained during the cultivation process.

After a 4 to 5 d cultivation and biotransformation process, the volume of pre-loaded 25 ml BMMY medium in a 500 ml flask drastically reduced through evaporation. Nonetheless, it was estimated that this integrated biodiesel production system contained 50% to 100% water relative to oil content. In previous immobilized enzyme- or whole cell lipase-based biodiesel production systems [22,23], water content was generally limited to very low level (<3%) to achieve satisfactory biodiesel yield through the addition of anhydrous molecular sieves or silica gels as water absorbent agents. The significantly enhanced water tolerance of this integrated system could significantly simplify the operation process and further reduce costs.

### Effect of methanol concentration on integrated biodiesel production via stepwise hydrolysis followed by esterification and reusability of this integrated system

Esterification involved in FAME production is an equilibrium reaction. Improving the methanol concentration drives this equilibrium towards FAME formation. However, a high concentration of methanol could deactivate lipases and deteriorate yeast cell growth in the integrated biodiesel production. Thus, the optimum methanol concentration is a compromise between minimizing lipase deactivation and maximizing the equilibrium balance towards FAME synthesis. The increased volume of water in the *in situ* reaction system allows more methanol to be present due to miscibility and prevention of insoluble methanol droplets, thereby reducing the deactivation of lipase by methanol droplets and hence facilitating biodiesel production. Also, for the intracellular lipase in the form of whole cell catalysts, the cell wall and membrane can provide a protective environment for lipases.

In this simultaneous lipase production and FAME synthesis system, we compared 8%, 14%, and 20% total methanol, corresponding to 3:1, 6:1, and 9:1 molar ratio to WCOs. To alleviate lipase deactivation and toxicity toward yeast cells, stepwise addition of methanol was adopted. Specifically, these varied amounts of total methanol were supplemented four times (that is, with 2%, 3.5%, or 5% methanol each time), every 12 h.

As shown in Figure 4B, 72%, 87%, and 41% biodiesel yield was achieved when 8%, 14%, and 20% total methanol was involved in biodiesel synthesis *in situ*, respectively. The highest biodiesel yield was derived using a ratio of 6:1 (methanol:oil), demonstrating better methanol tolerance in this integrated system than in other previous biocatalytic systems (generally 3:1 to 4:1) based on immobilized enzymes or whole cell catalysts [22,23]. Further increase of the amount of methanol (9:1) did not improve biodiesel production, likely because of the significant loss of lipase activity. Using *P. pastoris* whole cell catalysts with intracellular expression of the same Tll lipase with a lower water (5%) and methanol content (molar ratio of 4:1) in a solvent-free system resulted in a biodiesel yield of 82% [10]. By comparison, this integrated system, resisting higher contents of water and methanol (6:1), resulted in a higher biodiesel yield (87%). This could be attributed both to easily accessible extracellular lipase with high activity and the use of intracellular lipase in the form of whole cells for catalysis of biodiesel production.

Our investigation on the reusability of this integrated system showed 73%, 66%, and 61% relative biodiesel yield in three successive batches, respectively. The reusability of this integrated system is not as good as conventional immobilized enzyme or whole cell systems, because cell free extracellular enzymes in aqueous phase cannot be fully recovered as compared to immobilized particles or whole cells. We think the activity loss mainly comes from partial recovery, not lipase deactivation. However, the integrated system possesses a significant advantage in the overall cost of biodiesel production. Considering the savings on energy, and the cost for lipase immobilization or whole cell biocatalyst preparation versus traditional biodiesel production using separately prepared lipases, the *in situ* biodiesel synthesis process could reduce overall costs by approximately one-fourth compared with the conventional immobilized enzyme system.

## Conclusion

In this study, we proposed and investigated a new concept of integrated biodiesel production that combines lipase production and lipase-catalyzed biodiesel synthesis into a single integrated step. To achieve a high yield of biodiesel, two processes of integrated biodiesel production mediated by overexpressed Tll in *P. pastoris* yeast were compared: concurrent transesterification-esterification, and stepwise hydrolysis followed by esterification. The more efficient hydrolysis-esterification stepwise strategy gave rise to a 87% biodiesel yield under the optimized methanol concentration and water content. This finding demonstrates a promising biodiesel-producing methodology with a significantly simplified process and reduced cost, by skipping the costly step of enzyme isolation and preparation.

## Materials and methods

### Plasmid, strains, media, and reagents

The yeast host strain *P. pastoris* X33 and the plasmid pPICZαA were products of Invitrogen (Carlsbad, CA, USA). WCOs (FFA content of 19%, acid value of 170 mg KOH/g, saponification value of 366 mg KOH/g, density of 0.89 g/cm^3^, water content of 0.1%) were kindly donated by Lvming Environmental Protection Technology Co., Ltd. (Shanghai, China). n-hexadecane and authentic standards of FAMEs were purchased from Sigma-Aldrich (St. Louis, MO). The media including yeast extract peptone dextrose medium (YPDS), BMGY, BMMY, and low-salt lysogeny broth used in this study were prepared by following the manual of EasySelect™ *Pichia* Expression Kit (Invitrogen).

### Plasmid and recombinant strain construction

The codon optimized *tll* gene [GenBank:AF054513.1] was synthesized by GenScript Corporation and used as a PCR template. The primers (forward, *Eco*RI: 5′-CCG**GAATTC**TCACCTATCAGAAGAGAAGTTTCAC-3′ (the bold bases represent the *Eco*RI cutting site); (reverse, *Not*I: 5′-AAGGAA AAAA**GCGGCCGC**CTAATGATGATGATGATGATGCAAGCAAGTTCCGATAAG-3′ (the bold nucleotides indicate the *Not*I restriction site)) were used to amplify the *tll* gene fused with an artificial sequence encoding a His_6_-tag at the C-terminus of Tll lipase. The *Eco*RI and *Not*I double-digested *tll* fragment was inserted into the *Eco*RI/*Not*I pretreated pPICZαA, and the resultant pPICZαA-*tll* was transformed into *E. coli* DH5α competent cells. The sub-cloned *tll* sequence and its in-frame fusion with the *α* factor signal peptide was confirmed by DNA sequencing of the plasmids isolated from transformants grown on low-salt lysogeny broth medium containing 25 μg/ml zeocin. Then, pPICZαA-*tll* was linearized by *Sac*I and transformed into competent cells of *P. pastoris* X33 via electroporation. The positive transformants harboring chromosomal integration of Tll expression cassettes were preliminarily selected on YPDS plates supplemented with 100 μg/ml zeocin. Multi-copy integrated transformants were further screened on YPDS plates containing 2,000 μg/ml of zeocin.

### Expression of recombinant lipase

A single colony of *P. pastoris* harboring multi-copy Tll expression cassettes was used to inoculate 25 ml BMGY in a 500 ml baffled flask. Yeast cells were grown at 28°C, 250 rpm until optical cell density at a wavelength of 600 nm reaching 4 to 6. The cells were pelleted by centrifugation at room temperature under sterile conditions and re-suspended in a 500 ml baffled flask pre-loaded with 25 ml fresh BMMY medium (50 ml, 75 ml, or 100 ml). We added 0.5% (v/v) methanol every 12 h for induction of lipase expression. The strain containing empty vector pPICZαA was used as reference for the expression analysis.

### Measurement of cell free enzyme and whole cell activity

At designated time intervals, supernatant and cell pellet from 1 ml induced culture broth were separated by centrifugation, and used for measuring the hydrolytic activity of cell free enzymes and whole cell catalysts, respectively. The hydrolysis reactions using emulsified olive oil as the substrate were performed as previously described by Yan *et al*. [14], except that the reaction temperature was set at 40°C. The inactivated lipases in the form of cell free enzymes and whole cell catalysts were prepared by boiling samples at 100°C for 1 h and used as controls. One unit of lipase activity was defined as the amount of enzyme in 1 milliliter of supernatant or in 1 milligram of dry cells that hydrolyzes olive oil to form 1 μmol FFA per minute under testing conditions. Activity of cell free enzymes was presented as units of enzyme activity possessed by one milliliter of supernatant (U/ml). Activity of whole cell catalysts was calculated as units of enzyme activity possessed by one milligram of dry cell weight (U/mg DCW).

### Integrated lipase production and *in situ* biotransformation of waste cooking oils into biodiesel

At the post-induction time of 36 h (hereafter, unless otherwise specified, all times indicate post-induction time), 10 g WCOs were added into lipase-producing *P. pastoris* culture. In concurrent transesterification-esterification methanolysis, 2%, 3.5%, or 5% (v/v, methanol volume relative to culture volume) methanol was added at 36 h, 48 h, 60 h, and 72 h four times, resulting in the final molar ratio of WCOs to methanol at 1:3, 1:6, or 1:9, respectively. In the case of hydrolysis followed by esterification, 2%, 3.5%, or 5% (v/v) methanol was added at 48 h, 60 h, 72 h, and 84 h four times, allowing an extra 12 h for lipase-catalyzed hydrolysis in the absence of methanol. These *in situ* reactions were performed at 30°C and 250 rpm. The yeast cells harboring empty vector were subjected to the same expression procedure and reaction conditions were used as control.

For investigating reusability of this integrated system, the water layer containing extracellular enzymes and whole cells retaining intracellular enzymes were recovered by removing upper layer of FAMEs and residual oils. Glycerol adsorbed on whole cells was removed via washing. The recovered biocatalysts were applied into a new batch of biodiesel synthesis.

### Quantitative measurement of fatty acid methyl esters and free fatty acids

At certain time intervals, a 100 μl aliquot was withdrawn from the *in situ* reaction system and mixed with 100 μl deionized water, then centrifuged for 10 min at 11,000 × g. A 5 μl top layer was mixed with 995 μl n-hexane containing 1 mM n-hexadecane (internal standard) to prepare samples. A 1 μl sample was injected into Agilent 7890A GC system equipped with flame-ionization detector and a capillary column (INNOWAX, Agilent, Santa Clara, CA, 30 m × 0.25 mm × 0.25 μm) for FAME analysis. The gas chromatography oven was heated from 150°C to 260°C at a rate of 5°C/min, and kept at 260°C for 5 min. The injector and detector temperatures were 240°C and 280°C, respectively. The FAME content was quantified using the internal standard as reference, and calculated as percentage biodiesel yield relative to theoretical yield by following the previous report [11]. For measurement of FFA content, FFAs within a 100 μl oil phase in the reaction mixture were titrated by 50 mM NaOH using an autotitrator (ET 18, Mettler Toledo, Greifensee, Switzerland).

## Abbreviations

BMGY: buffered glycerol-complex medium; BMMY: buffered methanol-complex medium; DCW: dry cell weight; FAMEs: fatty acid methyl esters; FFAs: free fatty acids; Tll: *Thermomyces lanuginosus* lipase; WCOs: waste cooking oils.

## Competing interests

The authors declare that they have no competing interests.

## Authors’ contributions

JY and SL conceived of the study. JY and SL designed the experiments and prepared the manuscript. JY, XZ and LD performed the experiments, analyzed the data and helped to revise the manuscript. All authors read and approved the final manuscript.

## Supplementary Material

Additional file 1 **Graphical abstract.** Integrated lipase production and *in situ* biodiesel synthesis in a recombinant *Pichia pastoris* yeast: an efficient dual biocatalytic system composed of cell free enzymes and whole cell catalysts. Click here for file
