# Supplementary figures and images for: Integrated lipase production and in situ biodiesel synthesis in a recombinant Pichia pastoris yeast: an efficient dual biocatalytic system composed of cell free enzymes and whole cell catalysts
Source: Biotechnol Biofuels. 2014 Apr 8;7:55. doi: 10.1186/1754-6834-7-55 (PMC4022340; doi:10.1186/1754-6834-7-55)

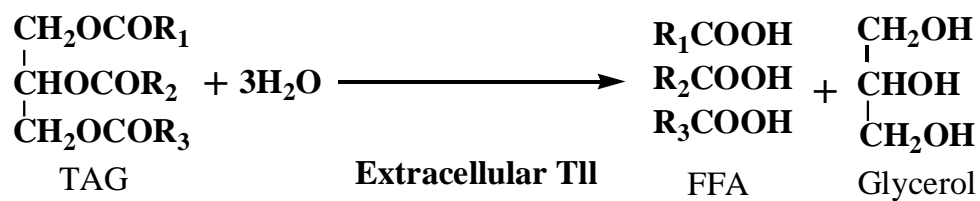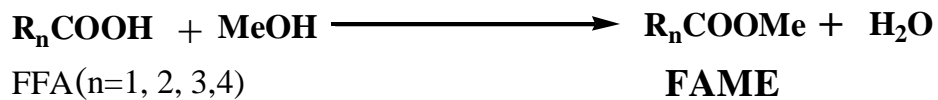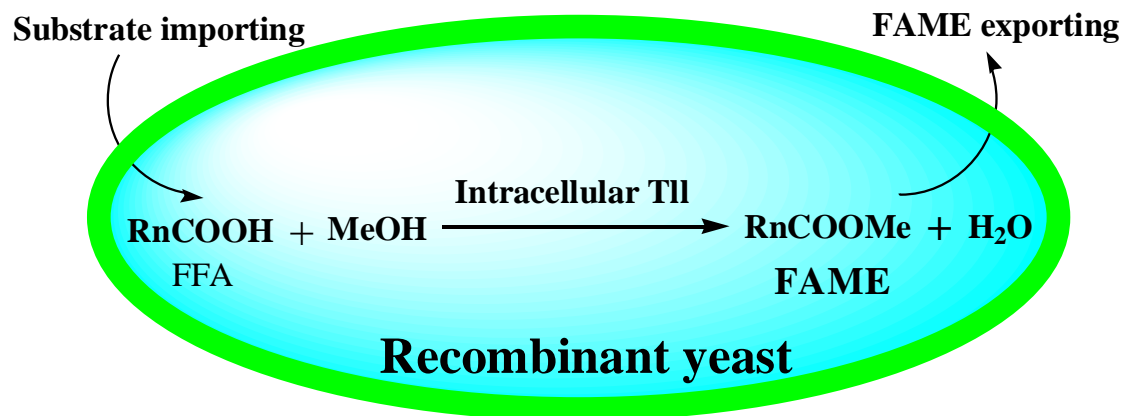

Supplement: Additional file 1 — Graphical abstract. Integrated lipase production and in situ biodiesel synthesis in a recombinant Pichia pastoris yeast: an efficient dual biocatalytic system composed of cell free enzymes and whole cell catalysts. [file 1754-6834-7-55-S1.pdf]
